# Does pathogen plasticity facilitate host shifts?

**DOI:** 10.1371/journal.ppat.1006961

**Published:** 2018-05-03

**Authors:** Henrik H. De Fine Licht

**Affiliations:** Section for Organismal Biology, Department of Plant and Environmental Sciences, University of Copenhagen, Frederiksberg, Denmark; University of Utah, UNITED STATES

**Fig 1:**
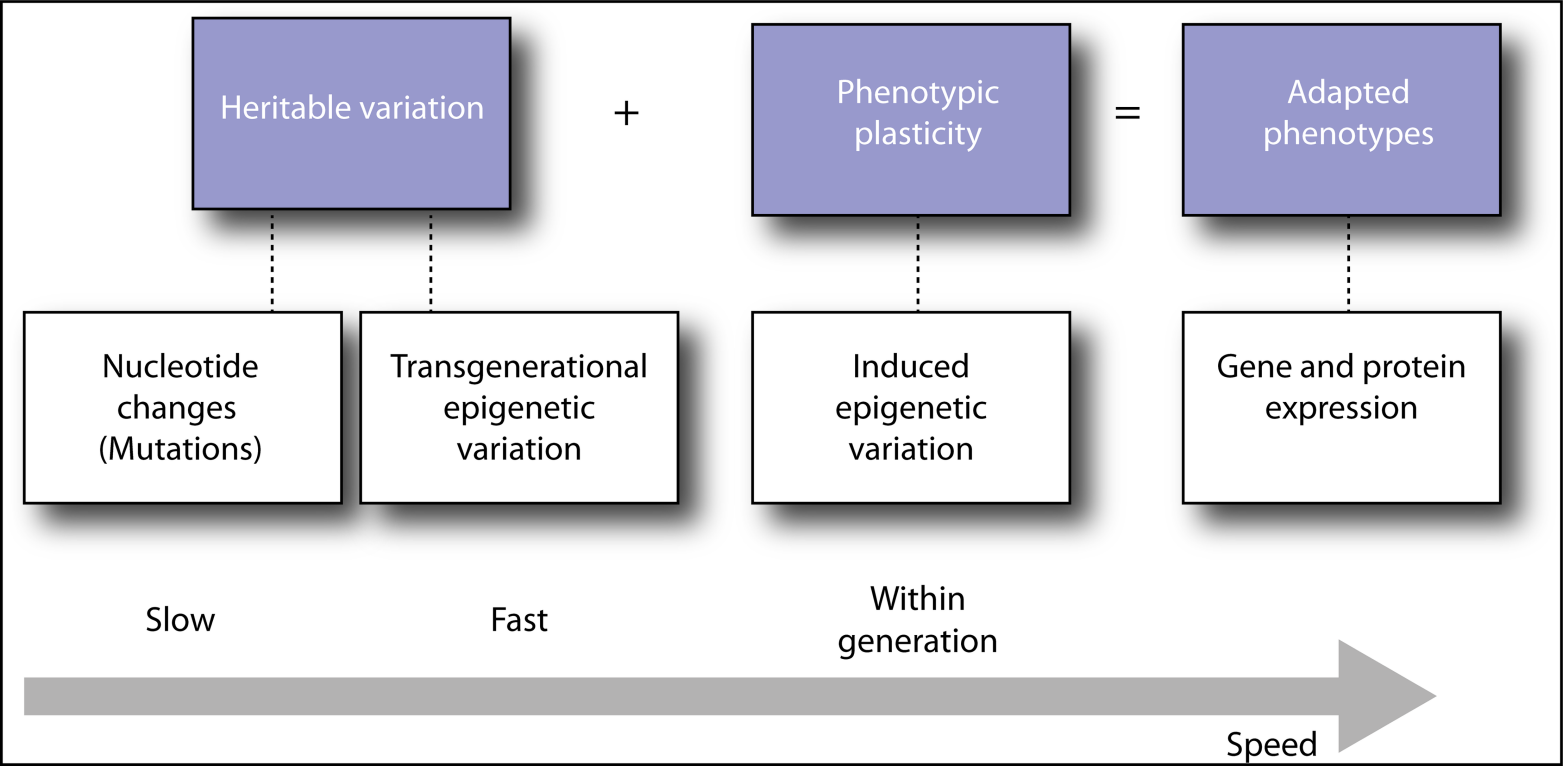
Heritable variation and phenotypic plasticity in combination shape–adapted phenotypes. Heritable variation consists of genetic variation and transgenerational epigenetic variation that differ in the rate at which changes occur. The most rapid changes occur in epigenetic variation that results in phenotypic plasticity within an organism’s lifetime, which generally are exempt from natural selection that only acts on heritable variation.

Pathogens that expand host range by shifting to a novel host taxon are a key factor for diversification and evolution of host–pathogen associations [1]. Such shifts are often also the initial spark for new emerging infectious diseases [2]. As a result, pathogen host shifts are of considerable concern for humans, wildlife, and agriculture, with obvious economic and public health impacts that threaten food biosecurity and human health [2,3]. Shifting to a new host may have a large impact on the evolution and genetic organization of the pathogen [4]. Indeed, many recent studies have investigated past and ongoing pathogen host shifts using genomic and population genetic methods [5–11], with much emphasis placed on characterizing the mutations, hybridizations, chromosomal reorganizations, or horizontal gene transfer events involved in host-shift genetics [12–14]. The rationale behind these studies is that such genomic changes often represent pathogen adaptation in response to the new environment of a new host. Because of the usually slow accumulation of mutational nucleotide changes (Fig 1), these genomic changes do not necessarily represent the factors responsible for facilitating the host shift in the first place. Instead, the extent to which a pathogen is able to adjust and produce a phenotype that can survive in the novel host, either via phenotypic plasticity [15,16] or cryptic genetic variation in the pathogen population [17–19], is increasingly recognized as an important driver for evolutionary innovation that can lead to niche expansion and pathogen host shifts [20–22].

**Fig 2:**
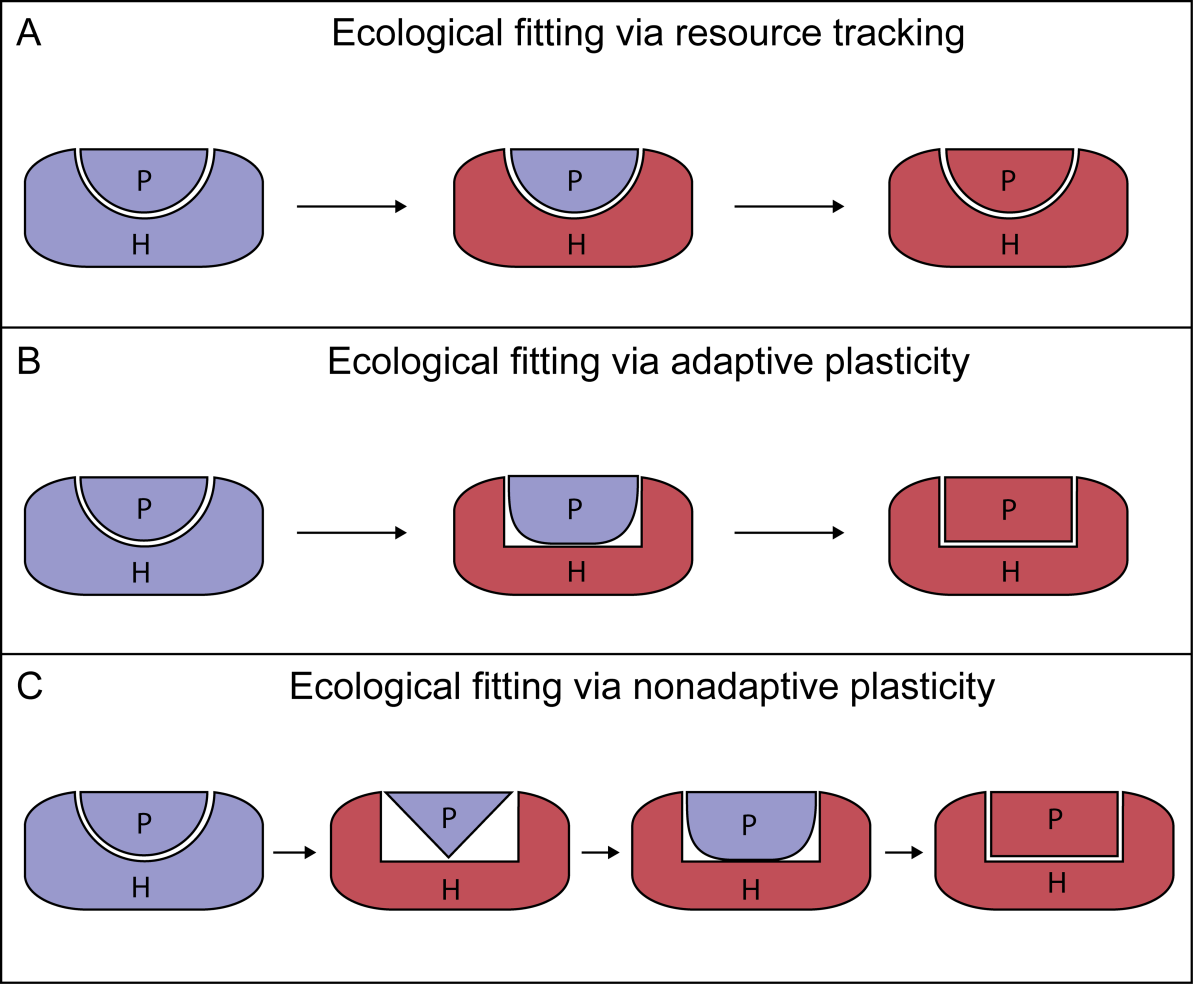
Scenarios of ecological fitting leading to pathogen host shifts. (A) The pathogen (blue P) is adapted to the native host (blue H), drawn as compatible pathogen and host shapes. During pathogen colonization of a new host (red H), the pathogen is readily able to infect the new host because of similarity in traits between the old and new host (compatible shapes between blue P and red H), which facilitates subsequent pathogen adaptation to the new host (red P and H). (B) When there is no similarity in traits between old (blue H) and new (red H) hosts, the pathogen cannot readily infect the new host. Instead, given that the pathogen changes phenotype via adaptive plasticity, it becomes more compatible with the new host (a close but not perfect match between shapes of blue P and red H). Such adaptive pathogen plasticity to previously unencountered host traits can thus facilitate subsequent pathogen adaptation to the new host (red P and H). (C) Similar to scenario B, there is no similarity in traits between old (blue H) and new (red H) hosts, and the pathogen cannot readily infect the new host. In this scenario, the pathogen changes phenotype via nonadaptive plasticity, which initially results in low compatibility between the pathogen (blue P) and the new host (red H). Such plastic responses with initially negative pathogen fitness on the novel host often expose hidden genetic variation to new regimes of natural selection. This genetic variation, previously shielded from natural selection, may then facilitate subsequent pathogen adaptation to the new host.

The process of host shifting involves several stages that each represent different ecological and evolutionary barriers for a new host–pathogen association to become established (Box 1). When ecological and spatial hindrances are overcome, pathogens are generally considered to shift hosts in either of two ways [21,23]. First, pathogens may colonize new hosts that represent a very similar resource to the ancestral host, i.e., ecological fitting via resource tracking (Fig 2A). This can occur if the original and new hosts are closely related or if the pathogen is exploiting traits that are evolutionarily conserved between the two host species. Hosts may be genetically diverse [24] or variably express the traits targeted by pathogens [25] so that only part of the new host population is susceptible at any given point in time. Second, pathogens may colonize new hosts that represent previously unencountered resources, i.e., ecological fitting via adaptive plasticity to host traits outside the range of conditions in which the pathogen evolved (Fig 2B). The completely novel and potentially stressful environment that a new host represents is, under this scenario, considered to “release” cryptic genetic variation in plasticity [26]. This variation in plastic responses leads to greater variation in pathogen phenotypes that provide the raw material for natural selection to shape the evolution of pathogenically relevant traits [20,27,28]. In cases where such plasticity produces an adaptive phenotype with improved fitness on the new host, it seems unproblematic to envisage how these novel and apparently “preadapted” pathogen phenotypes can eventually lead to a host shift (Fig 2B).

**Box 1. The biology of pathogen host shifts.**

The process of host shifts incorporates several steps. First, the pathogen must have the opportunity to shift hosts by exposure of the new host species to the pathogen. Many ecological barriers to transmission are breached by global trade, modern agricultural practices, and climate change, which facilitate more pathogen encounters and opportunities for infecting new potential hosts [68]. Second, the pathogen must be able to infect the new host. For example, viral pathogens use phylogenetically conserved receptors to infect host cells, and only hosts with appropriate cell receptors are compatible hosts [69]. The third and final step is for the pathogen to be sufficiently able to spread between individuals in the new host population. Between-host transmission is necessary for establishing the long-term associations characterizing a successful host shift in contrast to occasional spillover pathogen infections in the new host [70,71].

Here, I propose a third route for pathogenic host shifts that occur when the induced pathogen phenotypes on the new host are the result of nonadaptive plasticity (Fig 2C). Nonadaptive plasticity leads to an induced pathogen phenotype in the new host that, on average, has reduced fitness [26]. Nonadaptive plasticity may reflect a breakdown in an organism’s ability to maintain homeostasis and proper function, and this is usually considered to prevent pathogen host shifts from occurring [21,23,29]. However, similar to adaptive plastic responses, nonadaptive plasticity also exposes standing genetic variation to new regimes of natural selection. Evidence gathered to date, mainly from studies on interactions between nonpathogenic organisms, suggests that nonadaptive plasticity can have a major evolutionary impact and potentiate rapid adaptive evolution [28,30]. For example, in an experiment under natural conditions, wild guppy populations (*Poecilia reticulata*) were experimentally transplanted between streams with or without natural cichlid predators. After only 3 to 4 generations in the new environments, patterns of brain gene expression were shifted further away from the local optimum—i.e., nonadaptive plasticity—and potentiated adaptive evolution by increasing the strength of directional selection [30]. Nonadaptive plastic responses of pathogens undergoing a host shift therefore have the ability to further enhance the strong directional selection from the new host. Such pathogens will, however, initially have reduced fitness on the new hosts, which requires that the new host niche is initially not very competitive or that the new host compensates a lower rate of host exploitation by enhancing transmission. Furthermore, it is assumed that pathogen populations can persist for extended periods on suboptimal hosts, which is supported as a likely scenario in recent theoretical studies [29]. Finally, the new host population is assumed to vary in susceptibility to pathogen infection [24,31], which is characteristic of many host–pathogen systems and also supported by theoretical models [25].

**Fig 3:**
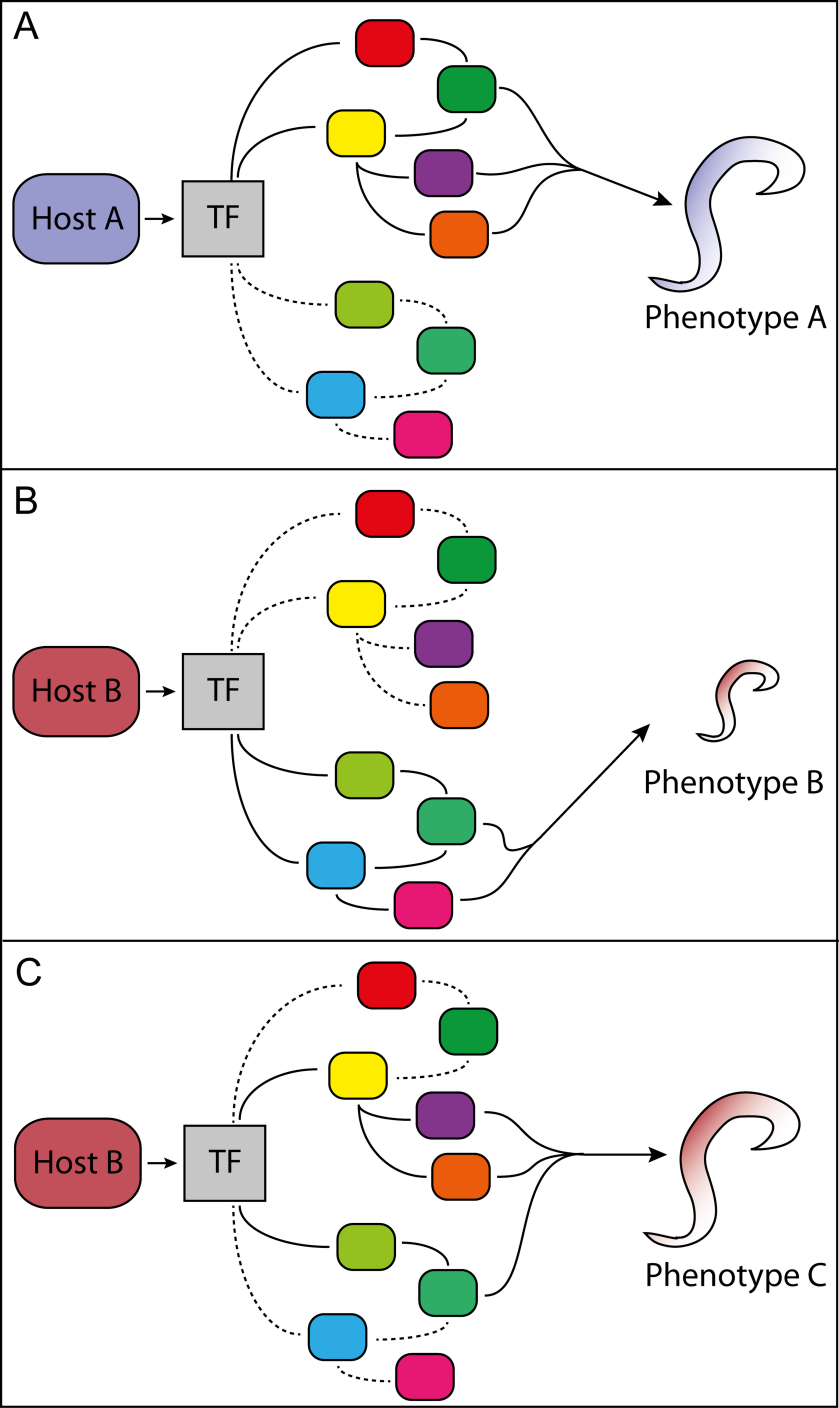
Illustration of how genetic assimilation of a nonadaptive plastic trait may be altered and fixed via partial co-option of GRNs. (A) The host-induced transcription factor (TF) controls a GRN of genes (different colored boxes), which leads to pathogen phenotype A (solid lines). Stippled lines connect genes in a GRN that is unused in this host and contains cryptic genetic variation. (B) A new host environment induces the TF to elicit previously unused modules of the GRN, which result in a nonadaptive plastic response (smaller size of phenotype B). (C) Provided phenotype B can survive long enough in host B, the old and new GRN modules may be co-opted into a new GRN, resulting in an adaptive phenotype C. GRN, gene regulatory network; TF, transcription factor.

Pathogens often display considerable phenotypic plasticity in response to changing environmental conditions in the host [32]. The expression of virulence traits may, for example, be contingent upon whether the host is infected with a single or multiple pathogens [33], and growth and size of pathogenic nematodes and trematodes can vary more than 10-fold, depending on infection intensity and host environment [34–37]. Pathogen plasticity may also be present as discrete phenotypes (polyphenisms), such as the lancet liver fluke *Dicrocoelium dendriticum*, in which a single cercaria usually positions itself against the subesophageal ganglion in the brain of the intermediate *Formica* spp. ant host, whereas the remaining cercariae develop into metacercariae in the gaster [38,39]. Plastic gene expression underlies phenotypic plasticity [40], which provides a way to measure subtle changes in phenotypic plasticity [41]. Recent methodological advances in dual-RNA sequencing (dual-RNAseq) analysis [42–44] allow changes in gene expression during host shifts to be monitored in many pathogen–host systems. Although transcriptome-wide datasets of gene expression are notoriously difficult to interpret, measuring changes in pathogen gene expression following host shifts provides a method to experimentally explore the role of nonadaptive plasticity for pathogen host shifts. This could, for example, be achieved by designing experiments that serially passage pathogens on novel hosts for multiple generations. Identifying genes that initially are differentially expressed on the new host but that, after passaging, change expression in the opposite direction would indicate nonadaptive expression. There is ample evidence that pathogens, such as the fungal human pathogens *Aspergillus fumigatus* and *Candida albicans* [45–49], adapt and change their gene repertoire in response to novel hosts or treatments. Similarly, the opportunistic human bacterial pathogen *Pseudomonas aeruginosa* employs plastic gene expression in response to variable infection conditions [50,51]. Even slight alterations of pathogen genes or transcription factors connected in gene regulatory networks (GRNs) may have a large evolutionary impact [15,52–54]. Such alteration of existing GRNs could be mediated by heritable epigenetic changes [55], and only need to involve partial or modular co-option of GRNs into new GRNs [56,57]. This would create an evolutionarily novel GRN combination that is exposed to strong directional selection in the new host and may eventually lead to a host shift (Fig 3).

Genome evolution differs fundamentally between eukaryotes on one hand and bacteria and viruses on the other, in which small gene-dense genomes, short generation times, and frequent horizontal gene exchange provide ample opportunity for new mutations to arise [10]. Populations of RNA viruses often contain extensive genetic diversity because of high mutation rates during RNA viral replication coupled with limited proofreading capacity [19]. The presence and generation of cryptic genetic diversity has been shown to be important in many RNA viral host shifts [13,58–60], for example, in avian influenza viruses by providing adaptive mutations in specific polymerase subunits that increase RNA polymerase activity in mammalian cells [61], or mutations that alter receptor binding to sialic acids or glycan linkages in mammalian cells [14]. Nonadaptive plasticity is considered to be of limited importance for RNA virus host shifts that are constrained and principally governed by genetic mutations [9,62] but is likely more important for host shifts influenced by variation in the conformation of RNA virus secondary structures that modulate interaction with the host immune system and increase persistence [63,64]. Eukaryotes contain larger and more plastic genomes, longer generation times, and sexual reproduction with recombination, which implies that rapid evolutionary adaptation is often governed by changes in gene expression and epigenetic markers instead of mutations that tend to emerge later [65] (Fig 1). Therefore, the importance of nonadaptive plasticity for mediating host shifts is likely higher in eukaryote pathogens such as pathogenic fungi, infectious worms, and trypanosome and malaria parasites than for bacterial and viral pathogens. Nonadaptive pathogen plasticity could help explain instances of extreme interkingdom host shifting [66] and the wide host range of some eukaryotic pathogens [67], which are not always easily explained by current host-shift models. However, more empirical work on how transcriptional, protein, and developmental networks in pathogens change in response to different host environments is required to understand the relative importance of adaptive versus nonadaptive plasticity for facilitating pathogen host shifts.
